# Certifying Certainty and Uncertainty in Approximate Membership Query Structures

**DOI:** 10.1007/978-3-030-53291-8_16

**Published:** 2020-06-16

**Authors:** Kiran Gopinathan, Ilya Sergey

**Affiliations:** 8 Microsoft Research Lab, Redmond, WA USA; 9 University of Southern California, Los Angeles, CA USA; 10 School of Computing, National University of Singapore, Singapore, Singapore; 11 Yale-NUS College, Singapore, Singapore

## Abstract

Approximate Membership Query structures (AMQs) rely on randomisation for time- and space-efficiency, while introducing a possibility of false positive and false negative answers. Correctness proofs of such structures involve subtle reasoning about bounds on probabilities of getting certain outcomes. Because of these subtleties, a number of unsound arguments in such proofs have been made over the years.

In this work, we address the challenge of building rigorous and reusable computer-assisted proofs about probabilistic specifications of AMQs. We describe the framework for systematic decomposition of AMQs and their properties into a series of interfaces and reusable components. We implement our framework as a library in the Coq proof assistant and showcase it by encoding in it a number of non-trivial AMQs, such as Bloom filters, counting filters, quotient filters and blocked constructions, and mechanising the proofs of their probabilistic specifications.

We demonstrate how AMQs encoded in our framework guarantee the absence of false negatives *by construction*. We also show how the proofs about probabilities of false positives for complex AMQs can be obtained by means of *verified reduction* to the implementations of their simpler counterparts. Finally, we provide a library of domain-specific theorems and tactics that allow a high degree of automation in probabilistic proofs.

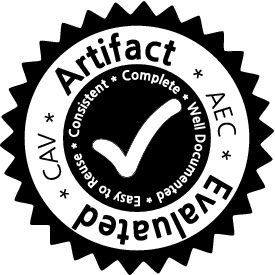



## Introduction

Approximate Membership Query structures (AMQs) are probabilistic data structures that compactly implement (multi-)sets via hashing. They are a popular alternative to traditional collections in algorithms whose utility is not affected by some fraction of wrong answers to membership queries. Typical examples of such data structures are Bloom filters [6], quotient filters [5, 38], and count-min sketches [12]. In particular, versions of Bloom filters find many applications in security and privacy [16, 18, 36], static program analysis [37], databases [17], web search [22], suggestion systems [45], and blockchain protocols [19, 43].

Hashing-based AMQs achieve efficiency by means of losing precision when answering queries about membership of certain elements. Luckily, most of the applications listed above can tolerate *some* loss of precision. For instance, a static points-to analysis may consider two memory locations as aliases even if they are not (a *false positive*), still remaining sound. However, it would be unsound for such an analysis to claim that two locations do not alias in the case they do (a *false negative*). Even if it increases the number of false positives, a randomised data structure can be used to answer aliasing queries in a sound way—as long as it does not have false negatives [37]. But *how much* precision would be lost if, *e.g.*, a Bloom filter with certain parameters is chosen to answer these queries? Another example, in which quantitative properties of false positives are critical, is the security of Bitcoin’s Nakamoto consensus [35] that depends on the counts of block production per unit time [19].

In the light of the described above applications, of particular interest are two kinds of properties specifying the behaviour of AMQs:

- *No-False-Negatives* properties, stating that a set-membership query for an element *x* always returns true if *x* is, in fact, in the set represented by the AMQ.
- Properties quantifying the rate of *False Positives* by providing a probabilistic bound on getting a wrong “yes”-answer to a membership query, given certain parameters of the data structure and the past history of its usage.

Given the importance of such claims for practical applications, it is desirable to have machine-checked formal proofs of their validity. And, since many of the existing AMQs share a common design structure, one may expect that a large portion of those validity proofs can be reused across different implementations.

Computer-assisted reasoning about the absence of *false negatives* in a particular AMQ (Bloom filter) has been addressed to some extent in the past [7]. However, to the best of our knowledge, mechanised proofs of probabilistic bounds on the *rates of false positives* did not extend to such structures. Furthermore, to the best of our knowledge, no other existing AMQs have been formally verified to date, and no attempts were made towards characterising the commonalities in their implementations in order to allow efficient proof reuse.

In this work, we aim to advance the state of the art in machine-checked proofs of probabilistic theorems about false positives in randomised hash-based data structures. As recent history demonstrates, when done in a “paper-and-pencil” way, such proofs may contain subtle mistakes [8, 10] due to misinterpreted assumptions about relations between certain kinds of events. These mistakes are not surprising, as the proofs often need to perform a number complicated manipulations with expressions that capture probabilities of certain events. Our goal is to factor out these reasoning patterns into a standalone library of *reusable* program- and specification-level definitions and theorems, implemented in a proof assistant enabling computer-aided verification of a variety of AMQs.

*Our Contributions. * The key novel observation we make in this work is the decomposition of the common AMQ implementations into the following components: (a) a hashing strategy and (b) a state component that operates over hash outcomes, together capturing most AMQs that provide fixed constant-time insertion and query operations. Any AMQ that is implemented as an instance of those components enjoys the *no-false-negatives* property *by construction*. Furthermore, such a decomposition streamlines the proofs of structure-specific bounds on false positive rates, while allowing for proof reuse for complex AMQ implementations, which are built on top of simpler AMQs [40]. Powered by those insights, this work makes the following technical contributions:

- A Coq-based mechanised framework Ceramist, specialised for reasoning about AMQs.^1^ Implemented as a Coq library, it provides a systematic decomposition of AMQs and their properties in terms of Coq modules and uses these interfaces to to derive certain properties “for free”, as well as supporting proof-by-reduction arguments between classes of similar AMQs.
- A library of non-trivial theorems for expressing closed-form probabilities on false positive rates in AMQs. In particular, we provide the first mechanised proof of the closed form for Stirling numbers of the second kind [26, Chap. 6].
- A collection of proven facts and tactics for effective construction of proofs of probabilistic properties. Our approach adopts the style of Ssreflect reasoning [21, 31], and expresses its core lemmas in terms of rewrites and evaluation.
- A number of case study AMQs mechanised via Ceramist: ordinary [6] and counting [46] Bloom filters, quotient filters [5, 38], and Blocked AMQs [40].

For ordinary Bloom filters, we provide the first mechanised proof that the probability of a false positive in a Bloom filter can be written as a closed form expression in terms of the input parameters; a bound that has often been mis-characterised in the past due to oversight of subtle dependencies between the components of the structure [6, 34]. For Counting Bloom filters, we provide the first mechanised proofs of several of their properties: that they have no false negatives, its false positive rate, that an element can be removed without affecting queries for other elements, and the fact that Counting Bloom filters preserve the number of inserted elements irrespective of the randomness of the hash outputs. For quotient filters, we provide a mechanised proof of the false positive rate and of the absence of false negatives. Finally, alongside the standard Blocked Bloom filter [40], we derive two novel AMQ data structures: *Counting Blocked Bloom filters* and *Blocked Quotient filters*, and prove corresponding no-false-negatives and false positive rates for all of them. Our case studies illustrate that Ceramist can be repurposed to verify hash-based AMQ structures, including entirely new ones that have not been described in the literature, but rather have been obtained by composing existing AMQs via the “blocked” construction.

Our mechanised development [24] is entirely *axiom-free*, and is compatible with Coq 8.11.0 [11] and MathComp  1.10 [31]. It relies on the infotheo library [2] for encoding discrete probabilities.

*Paper Outline.* We start by providing the intuition on Bloom filters, our main motivating example, in Sect. 2. We proceed by explaining the encoding of their semantics, auxiliary hash-based structures, and key properties in Coq in Sect. 3. Section 4 generalises that encoding to a general AMQ interface, and provides an overview of Ceramist, its embedding into Coq, showcasing it by another example instance—Counting Bloom filters. Section 5 describes the specific techniques that help to structure our mechanised proofs. In Sect. 6, we report on the evaluation of Ceramist on various case studies, explaining in detail our compositional treatment of blocked AMQs and their properties. Section 7 provides a discussion on the state of the art in reasoning about probabilistic data structures.

## Motivating Example

Ceramist is a library specialised for reasoning about AMQ data structures in which the underlying randomness arises from the interaction of one or more hashing operations. To motivate this development, we thus consider applying it to the classical example of such an algorithm—a Bloom filter [6].

### The Basics of Bloom Filters

Bloom filters are probabilistic data structures that provide compact encodings of mathematical sets, trading increased space efficiency for a weaker membership test [6]. Specifically, when testing membership for a value *not* in the Bloom filter, there is a possibility that the query may be answered as positive. Thus a property of direct practical importance is the exact probability of this event, and how it is influenced by the other parameters of the implementation.

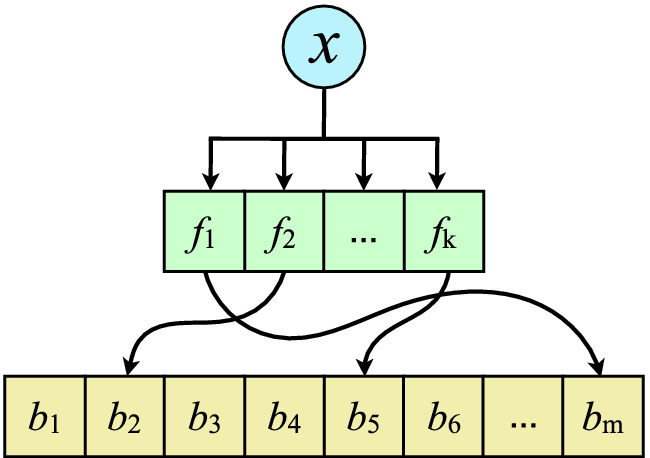



A Bloom filter $bf$ is implemented as a binary vector of *m* bits (all initially zeros), paired with a sequence of *k* hash functions $f_1, \dots , f_k$, collectively mapping each input value to a vector of *k* indices from $\left\{ {1 \ldots m}\right\}$, the indices determine the bits set to true in the *m*-bit array Assuming an ideal selection of hash functions, we can treat the output of $f_1, \dots , f_k$ on new values as a uniformly-drawn random vector. To insert a value *x* into the Bloom filter, we can treat each element of the “hash vector” produced from $f_1,\dots ,f_k$ as an index into $bf$ and set the corresponding bits to ones. Similarly, to test membership for an element *x*, we can check that all *k* bits specified by the hash-vector are raised.

### Properties of Bloom Filters

Given this model, there are two obvious properties of practical importance: that of false positives and of false negatives.

*False Negatives.* It turns out that these definitions are sufficient to guarantee the lack of false-negatives with complete certainty, *i.e.*, irrespective of the random outcome of the hash functions. This follows from the fact that once a bit is raised, there are no permitted operations that will unset it.

#### Theorem 1

**(No False Negatives).** If  $x~\in ~ bf$, then $\Pr \left[ x \in _? bf \right] = 1$, where $x \in _? bf$ stands for the approximate membership test, while the relation $x \in bf$ means that *x* has been previously inserted into $bf$.

*False Positives.* This property is more complex as the occurrence of a false positive is entirely dependent on the particular outcomes of the hash functions $f_1,\dots ,f_k$ and one needs to consider situations in which the hash functions happen to map some values to *overlapping* sets of indices. That is, after inserting a series of values $xs$, subsequent queries for $y \notin xs$ might incorrectly return true.

This leads to subtle dependencies that can invalidate the analysis, and have lead to a number of incorrect probabilistic bounds on the event, including in the analysis by Bloom in his original paper [6]. Specifically, Bloom first considered the probability that inserting *l* distinct items into the Bloom filter will set a particular bit $b_i$. From the independence of the hash functions, he was able to show that the probability of this event has a simple closed-form representation:

#### Lemma 1

**(Probability of a single bit being set).** If the only values previously inserted into $bf$ are $x_1,\dots ,x_l$, then the probability of a particular single bit at the position *i* being set is $\Pr \left[ i^{\mathrm{th}}\; \mathrm{bit\; in }\; bf \; \mathrm{is\; set}\right] =1 - \left( 1 - \frac{1}{m}\right) ^{kl}.$

Bloom then claimed that the probability of a false positive was simply the probability of a single bit being set, raised to the power of *k*, reasoning that a false positive for an element $y \not \in bf$ only occurs when all the *k* bits corresponding to the hash outputs are set.

Unfortunately, as was later pointed out by Bose *et al.* [8], as the bits specified by $f_1(x),\dots ,f_{k-1}(x)$ may overlap, we cannot guarantee the independence that is required for any simple relation between the probabilities. Bose *et al.* rectified the analysis by instead interpreting the bits within a Bloom filter as maintaining a set $\text {bits}( bf ) \subseteq \mathbb {N} _{[0,\dots ,m-1]}$, corresponding to the indices of raised bits. With this interpretation, an element *y* only tests positive if the random set of indices produced by the hash functions on *y* is such that $\text {inds}(y) \subseteq \text {bits}( bf )$. Therefore, the chance of a positive result for $y \not \in bf$ resolves to the chance that the random set of indices from hashing *y* is a subset of the union of $\text {inds}(x)$ for each $x \in bf$. The probability of this reduced event is described by the following theorem:

#### Theorem 2

**(Probability of False Positives).** If the only values inserted into $bf$ are $x_1,\dots ,x_l$, then for any $y \not \in bf$, $\Pr \left[ y \in _? bf \right] = \frac{1}{m^{k(l + 1)}} \sum _{i = 1}^{m} i^k i!$ $$\left( \begin{matrix} m \\ i \end{matrix} \right) \left\{ \begin{matrix} kl \\ i \end{matrix} \right\} , $$ where $$\left\{ \begin{matrix} s \\ t \end{matrix} \right\} $$ stands for the *Stirling number of the second kind*, capturing the number of surjections from a set of size *s* to a set of size *t*.

The key step in capturing these program properties is in treating the outcomes of hashes as *random variables* and then propagating this randomness to the results of the other operations. A formal treatment of program outcomes requires a suitable semantics, representing programs as distributions of such random variables. In moving to mechanised proofs, we must first fully characterise this semantics, formally defining a notion of a probabilistic computation in Coq.

## Encoding AMQs in Coq

To introduce our encoding of AMQs and their probabilistic behaviours in Coq, we continue with our running example, transitioning from mathematical notation to Gallina, Coq’s language. The rest of this section will introduce each of the key components of this encoding through the lens of Bloom filters.

### Probability Monad

Our formalisation represents probabilistic computations using an embedding following the style of the FCF library [39]. We do not use FCF directly, due to its primary focus on cryptographic proofs, wherein it provides little support for proving probabilistic bounds directly, instead prioritising a reduction-based approach of expressing arbitrary computations as compositions of known distributions.

Following the adopted FCF notation, a term of type  represents a probabilistic computation returning a value of type *A*, and is constructed using the standard monadic operators, with an additional primitive  that allows sampling from a uniform distribution over the range $\mathbb {Z}_{n}$: 
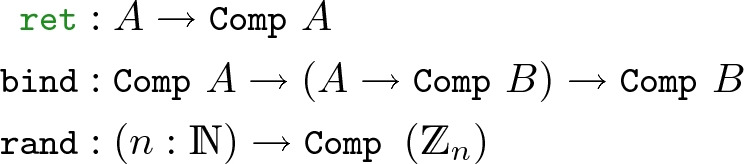


We implement a Haskell-style  -notation over this monad to allow descriptions of probabilistic computations within Gallina. For example, the following code is used to implement the query operation for the Bloom filter: 
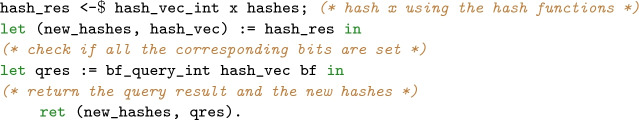
 In the above listing, we pass the queried value  along with the hash functions  to a probabilistic hashing operation  to hash  over each function in . The result of this random operation is then bound to  and split into its constituent components—a sequence of hash outputs  and an updated copy  of the hash functions, now incorporating the mapping for  . Then, having mapped our input into a sequence of indices, we can query the Bloom filter for membership using a corresponding deterministic operation  to check that all the bits specified by  are set. Finally, we complete the computation by returning the query outcome  and the updated hash functions  using the  operation to lift our result to a probabilistic outcome.

Using the code snippet above, we can define the query operation  as a function that maps a Bloom filter, a value to query, and a collection of hash functions to a probabilistic computation returning the query result and an updated set of hash functions. However, because our computation type does not impose any particular semantics, this result only encodes the *syntax* of the probabilistic query and has no actual meaning without a separate interpretation.

Thus, given a Gallina term of type , we must first evaluate it into a distribution over possible results to state properties on the probabilities of its outcomes. We interpret our monadic encoding in terms of Ramsey’s probability monad [42], which decomposes a complex distribution into composition of primitive ones bound together via conditional distributions. To capture this interpretation within Coq, we then use the encoding of this monad from the infotheo library [1, 2], and provide a function  that evaluates computations into distributions by recursively mapping them to the probability monad. Here,  represents infotheo ’s encoding of distributions over a finite support , defined as being composed of a measure function , and a proof that the sum of the measure over the support *A* produces 1.

This mapping from computations to distributions must be done to a program *e* (involving, *e.g.*, Bloom filter) before stating its probability bound. Therefore, we hide this evaluation process behind a notation that allows stating probabilistic properties in a form closer to their mathematical counterparts: 
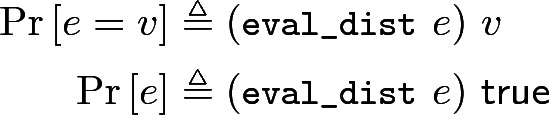


Above, *v* is an arbitrary element in the support of the distribution induced by *e*. Finally, we introduce a binding operator $\vartriangleright$ to allow concise representation of dependent distributions: .

### Representing Properties of Bloom Filters

We define the state of a Bloom filter (  ) in Coq as a binary vector of a fixed length *m*, using Ssreflect ’s  data type: 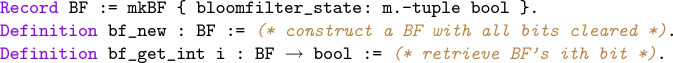 We define the deterministic components of the Bloom filter implementation as pure functions taking an instance of  and a series of indices assumed to be obtained from earlier calls to the associated hash functions:
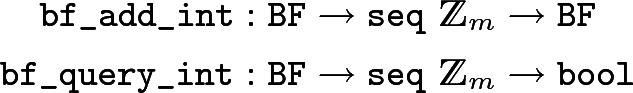


That is,  takes the Bloom filter state and a sequence of indices to insert and returns a new state with the requested bits also set. Conversely,  returns true *iff* all the queried indices are set. These pure operations are then called within a probabilistic wrapper that handles hashing the input and the book-keeping associated with hashing to provide the standard interface for AMQs: 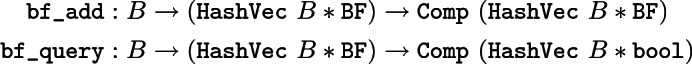


The component  (to be defined in Sect. 3.3), parameterised over an input type *B*, keeps track of *known results* of the involved hash functions and is provided as an external parameter to the function rather than being a part of the data structure to reflect typical uses of AMQs, wherein the hash operation is pre-determined and shared by *all* instances.

With these definitions and notation, we can now state the main theorems of interest about Bloom filters directly within Coq:^2^

#### Theorem 3

**(No False Negatives).** For any Bloom filter state $bf$, a vector of hash functions $hs$, after having inserted an element *x* into $bf$, followed by a series $xs$ of other inserted elements, the result of query $x \in _? bf$ is always true. That is, in terms of probabilities: 

#### Lemma 2

**(Probability of Flipping a Single Bit).** For a vector of hash functions $hs$ of length *k*, after inserting a series of *l* distinct values $xs$, all unseen in $hs$, into an empty Bloom filter $bf$, represented by a vector of *m* bits, the probability of its any index *i* being set is  Here,  is a simple embedding of the pure function  into a probabilistic computation.

#### Theorem 4

**(Probability of a False Positive).** After having inserted a series of *l* distinct values $xs$, all unseen in $hs$, into an empty Bloom filter $bf$, for any unseen $y \not \in xs$, the probability of a subsequent query $y \in _? bf$ for *y* returning true is given as 

The proof of this theorem required us to provide *the first axiom-free mechanised proof* for the closed form for Stirling numbers of the second kind [26].

In the definitions above, we used the output of the hashing operation as the bound between the deterministic and probabilistic components of the Bloom filter. For instance, in our earlier description of the Bloom filter query operation in Sect. 3.1, we were able to implement the entire operation with the only probabilistic operation being the call  . In general, structuring AMQ operations as manipulations with hash outputs via *pure* deterministic functions allows us to decompose reasoning about the data structure into a series of specialised properties about its deterministic primitives and a separate set of reusable properties on its hash operations.

### Reasoning About Hash Operations

We encode hash operations within our development using a random oracle-based implementation. In particular, in order to keep track of *seen* hashes learnt by hashing previously observed values, we represent a *state* of a hash function from elements of type  to a range $\mathbb {Z}_{m}$ using a finite map to ensure that previously hashed values produce the same hash output: 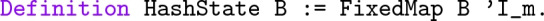 The state is paired with a hash function generating uniformly random outputs for unseen values, and otherwise returns the value as from its prior invocations: 
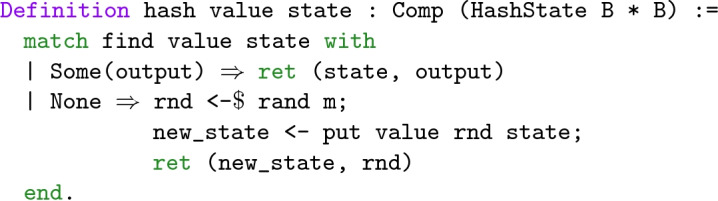
 A *hash vector* is a generalisation of this structure to represent a vector of states of *k* independent hash functions: 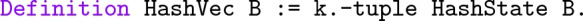 The corresponding hash operation over the hash vector, , is then defined as a function taking a value and the current hash vector and then returning a pair of the updated hash vector and associated random vector, internally calling out to  to compute individual hash outputs.

This random oracle-based implementation allows us to formulate several helper theorems for simplifying probabilistic computations using hashes by considering whether the hashed values *have been seen before or not*. For example, if we knew that a value *x* had not been seen before, we would know that the possibility of obtaining any particular choice of a vector of indices would be equivalent to obtaining the same vector by a draw from a corresponding uniform distribution. We can formalise this intuition in the form of the following theorem:

#### Theorem 5

**(Uniform Hash Output).** For any two hash vectors $$ hs $$, $$ hs '$$ of length *k*, a value *x* that has not been hashed before, and an output vector $$ {\iota }\!{s} $$ of length *m* obtained by hashing *x* via $$ hs $$, if the state of $$ hs '$$ has the same mappings as $$ hs $$ and also maps *x* to $$ {\iota }\!{s} $$, the probability of obtaining the pair $$( hs ', {\iota }\!{s} )$$ is uniform: .

Similarly, there are also often cases where we are hashing a value that we *have already seen*. In these cases, if we know the exact indices a value hashes to, we can prove a certainty on the value of the outcome:

#### Theorem 6

**(Hash Consistency).** For any hash vector $hs$, a value *x*, if $hs$ maps *x* to outputs ${\iota }\!{s}$, then hashing *x* again will certainly produce ${\iota }\!{s}$ and not change $hs$, that is, .

By combining these types of probabilistic properties about hashes with the earlier Bloom filter operations, we are able to prove the prior theorems about Bloom filters by reasoning primarily about the core logical interactions of the *deterministic components* of the data structure. This decomposition is not just applicable to the case of Bloom filters, but can be extended into a general framework for obtaining modular proofs of AMQs, as we will show in the next section.

## Ceramist at Large

Zooming out from the previous discussion of Bloom filters, we now present Ceramist in its full generality, describing the high-level design in terms of the various interfaces it requires to instantiate to obtain verified AMQ implementations.

The core of our framework revolves around the decomposition of an AMQ data structure into separate interfaces for hashing (AMQHash) and state (AMQ), generalising the specific decomposition used for Bloom filters (hash vectors and bit vectors respectively). More specifically, the AMQHash interface captures the probabilistic properties of the hashing operation, while the AMQ interface captures the deterministic interactions of the state with the hash outcomes.

### AMQHash Interface

The AMQHash interface generalises the behaviours of hash vectors (Sect. 3.3) to provide a generic description of the hashing operation used in AMQs.

The interface first abstracts over the specific types used in the prior hashing operations (such as, *e.g.*,  ) by treating them as opaque parameters: using a parameter  to represent the state of the hash operation; types  and  encoding the hash inputs and outputs respectively, and finally, a deterministic operation  to encode the interaction of the state with the outputs and inputs. For example, in the case of a single hash, the state parameter  would be  , while for a hash vector this would instead be  .

To use this hash state in probabilistic computations, the interface assumes a separate probabilistic operation that will take the hash state and randomly generate an output (*e.g.*,  for single hashes and  for hash vectors): 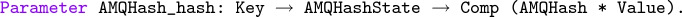 Then, to abstractly capture the kinds of reasoning about the outcomes of hash operations done with Bloom filters in Sect. 3.3, the interface assumes a few predicates on the hash state to provide information about its contents: 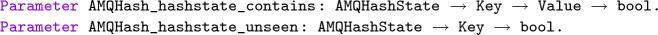 These components are then combined together to produce more abstract formulations of the previous Theorems 5 and 6 on hash operations.

#### Property 1

**(Generalised Uniform Hash Output).** There exists a probability $$p_{\text {hash}}$$, such that for any two AMQ hash states $$ hs , hs '$$, a value *x* that is unseen, and an output $$ {\iota }\!{s} $$ obtained by hashing *x* via $$ hs $$, if the state of $$ hs '$$ has the same mappings as $$ hs $$ and also maps *x* to $$ {\iota }\!{s} $$, the probability of obtaining the pair $$( hs ', {\iota }\!{s} )$$ is given by: .

#### Property 2

**(Generalised Hash Consistency).** For any AMQ hash state $hs$, a value *x*, if $hs$ maps *x* to an output ${\iota }\!{s}$, then hashing *x* again will certainly produce ${\iota }\!{s}$ and not change $hs$: 

Proofs of these corresponding properties must also be provided to instantiate the AMQHash interface. Conversely, components operating over this interface can assume their existence, and use them to abstractly perform the same kinds of simplifications as done with Bloom filters, resolving many probabilistic proofs to dealing with deterministic properties on the AMQ states.

### The AMQ Interface

Building on top of an abstract AMQHash component, the AMQ interface then provides a unified view of the state of an AMQ and how it deterministically interacts with the output type  of a particular hashing operation.

As before, the interface begins by abstracting the specific types and operations of the previous analysis of Bloom filters, first introducing a type  to capture the state of the AMQ, and then assuming deterministic implementations of the typical *add* and *query* operations of an AMQ: 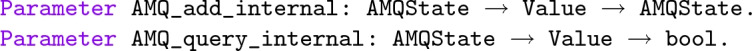 In the case of Bloom filters, these would be instantiated with the  ,  and  operations respectively (*cf.*  Sect. 3.2), thereby setting the associated hashing operation to the hash vector (Sect. 3.3).

As we move on to reason about the behaviours of these operations, the interface diverges slightly from that of the Bloom filter by conditioning the behaviours on the assumption that the state has sufficient capacity: 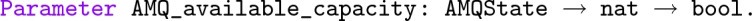 While the Bloom filter has no real deterministic notion of a capacity, this cannot be said of all AMQs in general, such as the Counting Bloom filter or Quotient filter, as we will discuss later.

With these definitions in hand, the behaviours of the AMQ operations are characterised using a series of associated assumptions:

#### Property 3

**(AMQ insertion validity).** For a state *s* with sufficient capacity, inserting any hash output ${\iota }\!{s}$ into *s* via  will produce a new state $s'$ for which any subsequent queries for ${\iota }\!{s}$ via  will return true.

#### Property 4

**(AMQ query preservation).** For any AMQ state *s* with sufficient remaining capacity, if queries for a particular hash output ${\iota }\!{s}$ in *s* via  happen to return true, then inserting any further outputs ${\iota }\!{s} '$ into *s* will return a state for which queries for ${\iota }\!{s}$ will *still* return true.

Even though these assumptions seemingly place strict restrictions on the permitted operations, we found that these properties are satisfied by most common AMQ structures. One potential reason for this might be because they are in fact *sufficient* to ensure the No-False-Negatives property standard of most AMQs:

#### Theorem 7

**(Generalised No False Negatives).** For any AMQ state *s*, a corresponding hash state $hs$, after having inserted an element *x* into *s*, followed by a series $xs$ of other inserted elements, the result of query for *x* is always true. That is, 

Here,  ,  , and  are generalisations of the probabilistic wrappers of Bloom filters (*cf.*  Sect. 3.1) for doing the bookkeeping associated with hashing and delegating to the internal deterministic operations.

The generalised Theorem 7 illustrates one of the key facilities of our framework, wherein by simply providing components satisfying the AMQHash and AMQ interfaces, it is possible to obtain proofs of certain standard probabilistic properties or simplifications *for free*.

**Fig. 1.**
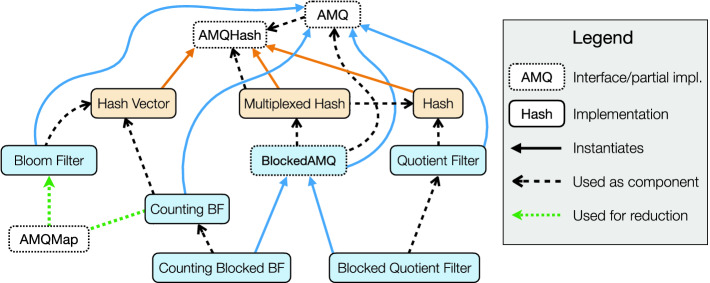
Overview of Ceramist and dependencies the between its components.

The diagram in Fig. 1 provides a high-level overview of the interfaces of Ceramist, their specific instances, and dependencies between them, demonstrating Ceramist ’s take on compositional reasoning and proof reuse. For instance Bloom filter implementation instantiates the AMQ interface implementation and uses, as a component, hash vectors, which themselves instantiate AMQHash used by AMQ. Bloom filter itself is also used as a proof reduction target by Counting Bloom filter. We will elaborate on this and the other noteworthy dependencies between interfaces and instances of Ceramist in the following sections.

### Counting Bloom Filters Through Ceramist

To provide a concrete demonstration of the use of the AMQ interface, we now switch over to a new running example—Counting Bloom filters [46]. A Counting Bloom filter is a variant of the Bloom filter in which individual bits are replaced with counters, thereby allowing the removal of elements. The implementation of the structure closely follows the Bloom filter, generalising the logic from bits to counters: insertion increments the counters specified by the hash outputs, while queries treat counters as set if greater than 0. In the remainder of this section, we will show how to encode and verify the Counting Bloom filter for the standard AMQ properties. We have also proven two novel domain-specific properties of Counting Bloom filters (*cf.* Appendix A of the extended paper version [25]).

First, as the Counting Bloom filter uses the same hashing strategy as the Bloom filter, the hash interface can be instantiated with the Hash Vector structure used for the Bloom filter, entirely reusing the earlier proofs on hash vectors. Next, in order to instantiate the AMQ interface, the state parameter can be defined as a vector of bounded integers, all initially set to 0: 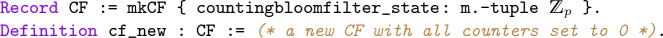 As mentioned before, the *add* operation increments counters rather than setting bits, and the *query* operation treats counters greater than 0 as raised.
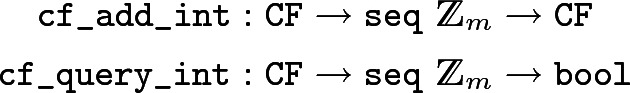


To prevent integer overflows, the counters in the Counting Bloom filter are bounded to some range $\mathbb {Z}_{p}$, so the overall data structure too has a maximum capacity. It would not be possible to insert any values if doing such would raise any of the counters above their maximum. To account for this, the capacity parameter of the AMQ interface is instantiated with a simple predicate  that verifies that the structure can support *l* further inserts by ensuring that each counter has at least $k * l$ spaces free (where *k* is the number of hash functions used by the data structure).

The add operation can be shown to be monotone on the value of any counter when there is sufficient capacity (Property 3). The remaining properties of the operations also trivially follow, thereby completing the instantiation, and allowing the automatic derivation of the No-False-Negatives result via Theorem 7.

### Proofs About False Positive Probabilities by Reduction

As the observable behaviour of Counting Bloom filter almost exactly matches that of the Bloom filter, it seems reasonable that the same probabilistic bounds should also apply to the data structure. To facilitate these proof arguments, we provide the AMQMap interface that allows the derivation of probabilistic bounds by reducing one AMQ data structure to another.

The AMQMap interface is parameterised by two AMQ data structures, AMQ A and B, using the same hashing operation. It is assumed that corresponding bounds on False Positive rates have already been proven for AMQ B, while have not for AMQ A. The interface first assumes the existence of some mapping from the state of AMQ A to AMQ B, which satisfies a number of properties: 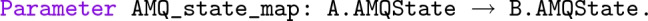


In the case of our Counting Bloom filter example, this mapping would convert the Counting Bloom filter state to a bit vector by mapping each counter to a raised bit if its value is greater than 0. To provide the of the false positive rate boundary, the AMQMap interface then requires the behaviour of this mapping to satisfy a number of additional assumptions:

#### Property 5

**(AMQ Mapping Add Commutativity).** Adding a hash output to the AMQ B obtained by applying the mapping to an instance of AMQ A produces the same result as first adding a hash output to AMQ A and then applying the mapping to the result.

#### Property 6

**(AMQ Mapping Query Preservation).** Applying B’s query operation to the result of mapping an instance of AMQ A produces the same result as applying A’s query operation directly.

In the case of reducing Counting Bloom filters (A) to Bloom filters (B), both results follow from the fact that after incrementing the some counters, all of them will have values greater than 0 and thus be mapped to raised bits.

Having instantiated the AMQMap interface with the corresponding function and proofs about it, it is now possible to derive the false positive rate of Bloom filters for Counting Bloom filters for free through the following generalised lemma:

#### Theorem 8

**(AMQ False Positive Reduction).** For any two AMQs A, B, related by the AMQMap interface, if the false positive rate for B after inserting *l* items is given by the function *f* on *l*, then the false positive rate for *A* is also given by *f* on *l*. That is, in terms of probabilities: 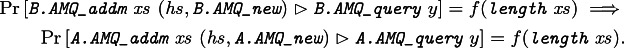


## Proof Automation for Probabilistic Sums

We have, until now, avoided discussing details of how facts about the probabilistic computations can be composed, and thereby also the specifics of how our proofs are structured. As it turns out, most of this process resolves to reasoning about summations over real values as encoded by Ssreflect ’s bigop library. Our development also relies on the tactic library by Martin-Dorel and Soloviev [32].

In this section, we outline some of the most essential proof principles facilitating the proofs-by-rewriting about probabilistic sums. While most of the provided rewriting primitives are standalone general equality facts, some of our proof techniques are better understood as combining a series of rewritings into a more general rewriting pattern. To delineate these two cases, will use the terminology **Pattern** to refer to a general pattern our library supports by means of a dedicated Coq tactic, while **Lemma** will refer to standalone proven equalities.

### The Normal Form for Composed Probabilistic Computations

When stating properties on outcomes of a probabilistic computation (*cf.*  Sect. 3.1), the computation must first be recursively evaluated into a distribution, where the intermediate results are combined using the probabilistic  operator. Therefore, when decomposing a probabilistic property into smaller subproofs, we must rely on its semantics that is defined for discrete distributions as follows:
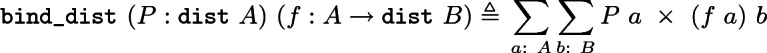


Expanding this definition, one can represent any statement on the outcome of a probabilistic computation in a *normal form* composed of only nested summations over a product of the probabilities of each intermediate computational step. This paramount transformation is captured as the following pattern:

#### Pattern 1

**(Bind normalisation)**

$$\begin{aligned} \Pr \left[ (c_1 \vartriangleright \ldots \vartriangleright c_m) = v \right] = \sum _{v_1} \cdots \sum _{v_{m - 1}} \Pr \left[ c_1 = v_1 \right] \times \cdots \times \Pr \left[ c_m~v_{m-1} = v \right] \end{aligned}$$

Here, by $c_i~v_{i-1} = v_i$, we denote the event in which the result of evaluating the command $c_i~v_{i - 1}$ is $v_i$, where $v_{i - 1}$ is the result of evaluating the previous command in the chain. This transformation then allows us to resolve the proof of a given probabilistic property into proving simpler statements on its substeps. For instance, consider the implementation of Bloom filter’s query operation from Sect. 3.1. When proving properties of the result of a particular query (as in Theorem 3), we use this rule to decompose the program into its component parts, namely as being the product of a hash invocation  and the deterministic query operation . This allows dealing with the hash operation and the deterministic component *separately* by applying subsequent rewritings to each factor on the right-hand side of the above equality.

### Probabilistic Summation Patterns

Having resolved a property into our normal form via a tactic implementing Pattern 1, the subsequent reductions rely on the following patterns and lemmas.

*Sequential Composition.* When reasoning about the properties of composite programs, it is common for some subprogram *e* to return a probabilistic result that is then used as the arguments for a probabilistic function *f*. This composition is encapsulated by the operation $e \vartriangleright f$ , as used by Theorems 3, 2, and 4. The corresponding programs, once converted to the normal form, are characterised by having factors within its internal product that simply evaluate the probability of the final statement  to produce a particular value $v_k$:
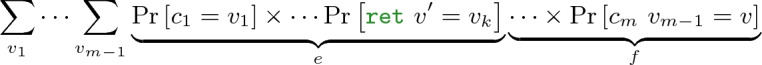


Since the return operation is defined as a delta distribution with a peak at the return value $v'$, we can simplify the statement by removing the summation over $v_k$, and replacing all occurrences of $v_k$ with $v'$, via the following pattern:

#### Pattern 2

**(Probability of a Sequential Composition).**
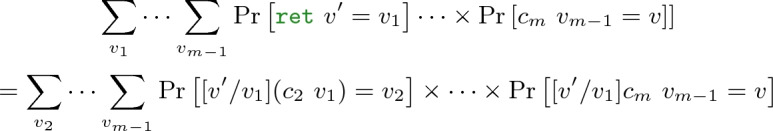


Notice that, without loss of generality, Pattern 2 assumes that the $v'$-containing factor is in the head. Our tactic implicitly rewrites the statement to this form.

*Plausible Statement Sequencing.* One common issue with the normal form, is that, as each statement is evaluated over the entirety of its support, some of the dependencies between statements are obscured. That is, the outputs of one statement may in fact be constrained to *some subset* of the complete support. To recover these dependencies, we provide the following theorem, that allows reducing computations under the assumption that their inputs are plausible:

#### Lemma 3

**(Plausible Sequencing).** For any computation sequence $c_1 \vartriangleright c_2$, if it is possible to reduce the computation $c_2 ~ x$ to a simpler form $c_3 ~ x$ when *x* is amongst plausible outcomes of $c_1$, (*i.e.*, $\Pr \left[ c_1 = x\right] \ne 0$ holds) then it is possible to rewrite $c_2$ to $c_3$ without changing the resulting distribution:

$$ \sum _{x} \sum _{y} \Pr \left[ c_1 = x\right] \times \Pr \left[ c_2 ~ x = y\right] = \sum _{x} \sum _{y} \Pr \left[ c_1 = x\right] \times \Pr \left[ c_3 ~ x = y\right] $$

*Plausible Outcomes.* As was demonstrated in the previous paragraph, it is sometimes possible to gain knowledge that a particular value *v* is a plausible outcome for a composite probabilistic computation $c_1 \vartriangleright \dots \vartriangleright c_m$:

$$\begin{aligned} \sum _{v_1} \cdots \sum _{v_{m - 1}} \Pr \left[ c_1 = v_1 \right] \times \cdots \times \Pr \left[ c_m~v_{m-1} = v \right] \ne 0 \end{aligned}$$

This fact in itself is not particularly helpful as it does not immediately provide any usable constraints on the value *v*. However, we can now turn this inequality into a conjunction of inequalities for individual probabilities, thus getting more information about the intermediate steps of the computation:

#### Pattern 3

If  $\sum _{v_1} \cdots \sum _{v_{m - 1}} \Pr \left[ c_1 = v_1 \right] \times \cdots \times \Pr \left[ c_m~v_{m-1} = v \right] \ne 0,$ then there exist $v_1, \dots , v_{m-1}$ such that $\Pr \left[ c_1 = v_1 \right] \ne 0 \wedge \cdots \wedge \Pr \left[ c_m = v \right] \ne 0.$

This transformation is possible due to the fact that probabilities are always non-negative, thus if a summation is positive, there must exist at least one element in the summation that is also positive.

*Summary of the Development.* By composing these components together, we obtain a comprehensive toolbox for effectively reasoning about probabilistic computations. We find that our summation patterns end up encapsulating most of the book-keeping associated with our encoding of probabilistic computations, which, combined with the AMQ/AMQHash decomposition from Sect. 4, allows for a fairly straightforward approach for verifying properties of AMQs.

### A Simple Proof of Generalised No False Negatives Theorem

To showcase the fluid interaction of our proof principles in action, let us consider the proof of the generalised No-False-Negatives Theorem 7, stating the following:1As with most of our probabilistic proofs, we begin by applying normalisation Pattern 1 to reduce the computation into our normal form: 
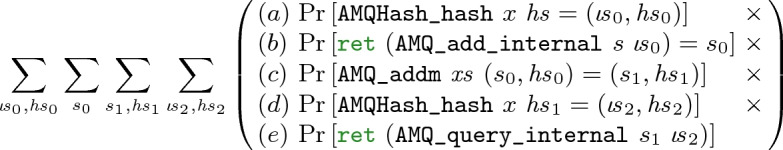
 We label the factors to be rewritten as (*a*)–(*e*) for the convenience of the presentation, indicating the correspondence to the components of the statement (1). From here, as all values are assumed to be unseen, we can use Property 1 in conjunction with the sequencing Pattern 2 to reduce factors (*a*) and (*b*) as follows:
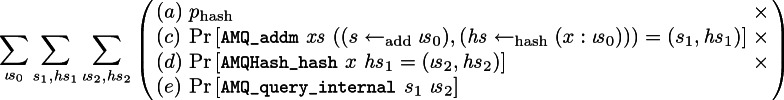


Here, $$p_{\text {hash}}$$ is the probability from the statement of Property 1. We also introduce the notations $$s \leftarrow _{\text {add}} {\iota }\!{s} _0$$ and $$ hs \leftarrow _{\text {hash}}(x: {\iota }\!{s} _0)$$ to denote the deterministic operations  and  respectively. Then, using Pattern 3 for decomposing plausible outcomes, it is possible to separately show that any plausible $$ hs _1$$ from  must map *x* to $$ {\iota }\!{s} _0$$, as hash operations preserve mappings. Combining this fact with Lemma 3 (plausible sequencing) and Hash Consistency (Property 2), we can derive that the execution of  on *x* in (*d*) must return $$ {\iota }\!{s} _0$$, simplifying the summation even further: 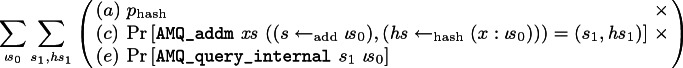


Finally, as $s_1$ is a plausible outcome from  called on $s \leftarrow _{\text {add}} {\iota }\!{s} _0$, we can then show, using Property 4 (query preservation), that querying for ${\iota }\!{s} _0$ on $s_1$ must succeed. Therefore, the entire summation reduces to the summation of distributions over their support, which can be trivially shown to be 1.

## Overview of the Development and More Case Studies

The Ceramist mechanised framework is implmented as library in Coq proof assistant [24]. It consists of three main sub-parts, each handling a different aspect of constructing and reasoning about AMQs: (*i*) a library of *bounded-length data structures*, enhancing MathComp ’s [31] support for reasoning about finite sequences with varying lengths; (*ii*) a library of *probabilistic computations*, extending the infotheo probability theory library [2] with definitions of deeply embedded probabilistic computations and a collection of tactics and lemmas on summations described in Sect. 5; and (*iii*) the *AMQ interfaces and instances* representing the core of our framework described in Sect. 4.

Alongside these core components, we also include four specific case studies to provide concrete examples of how the library can be used for practical verification. Our first two case studies are the mechanisation of the Bloom filter [6] and the Counting Bloom filter [46], as discussed earlier. In proving the false-positive rate for Bloom filters, we follow the proof by Bose *et al.* [8], also providing the first mechanised proof of the closed expression for Stirling numbers of the second kind. Our third case study provides mechanised verification of the quotient filter [5]. Our final case study is a mechanisation of the Blocked AMQ—a family of AMQs with a common aggregation strategy. We instantiate this abstract structure with each of the prior AMQs, obtaining, among others, a mechanisation of Blocked Bloom filters [40]. The sizes of each library component, along with the references to the sections that describe them, are given in the table above.

**Table:** 

Section	Size (LOC)	
	Specifications	Proofs
Bounded containers	286	1051
Notation (Sect. 3.1)	77	0
Summations (Sect. 5)	742	2122
Hash operations (Sect. 4.1)	201	568
AMQ framework (Sect. 4.2)	594	695
Bloom filter (Sect. 3.2)	322	1088
Counting BF (Sect. 4.4, [25, Sect. A])	312	674
Quotient filter (Sect. 6.1)	197	633
Blocked AMQ (Sect. 6.2)	269	522

Of particular note, in effect due to the extensive proof reuse supported by Ceramist, the proof size for each of our case-studies *progressively decreases*, with around a 50% reduction in the size from our initial proofs of Bloom filters to the final case-studies of different Blocked AMQs instances.

### Quotient Filter

A quotient filter [5] is a type of AMQ data structure optimised to be more cache-friendly than other typical AMQs. In contrast to the relatively simple internal vector-based states of the Bloom filters, a quotient filter works by internally maintaining a hash table to track its elements.

The internal operations of a quotient filter build upon a fundamental notion of *quotienting*, whereby a single *p*-bit hash outcome is split into two by treating the upper *q*-bits (the quotient) and the lower *r*-bits (the remainder) separately. Whenever an element is inserted or queried, the item is first hashed over a single hash function and then the output quotiented. The operations of the quotient filter then work by using the *q*-bit quotient to specify a bucket of the hash table, and the *r*-bit remainder as a proxy for the element, such that a query for an element will succeed if its remainder can be found in the corresponding bucket.

A false positive can occur if the outputs of the hash function happen to exactly collide for two particular values (collisions in just the quotient or remainder are not sufficient to produce an incorrect result). Therefore, it is then possible to reduce the event of a false positive in a quotient filter to the event that at least one in several draws from a uniform distribution produces a particular value. We encode quotient filters by instantiating the AMQHash interface from Sect. 4.1 with a *single* hash function, rather than a vector of hash functions, which is used by the Bloom filter variants (Sect. 2). The size of the output of this hashing operation is defined to be $2^q * 2^r$, and a corresponding quotienting operation is defined by taking the quotient and remainder from dividing the hash output by $2^q$. With this encoding, we are able to provide a mechanised proof of the false positive rate for the quotient filter implemented using *p*-bit hash as being:

#### Theorem 9

**(Quotient filter False Positive Rate).** For a hash-function $hs$, after inserting a series of *l* unseen distinct values $xs$ into an empty quotient filter $qf$, for any unseen $y \not \in xs$, the probability of a query $y \in _? qf$ for *y* returning true is given by: 

### Blocked AMQ

Blocked Bloom filters [40] are a cache-efficient variant of Bloom filters where a single instance of the structure is composed of a vector of *m* independent Bloom filters, using an additional “meta”-hash operation to distribute values between the elements. When querying for a particular element, the meta-hash operation would first be consulted to select a particular instance to delegate the query to.

While prior research has only focused on applying this blocking design to Bloom filters, we found that this strategy is in fact generic over the choice of AMQ, allowing us to formalise an abstract Blocked AMQ structure, and later instantiate it for particular choices of “basic” AMQs. As such, this data structure highlights the scalability of Ceramist *wrt.* composition of programs and proofs.

Our encoding of Blocked AMQs within Ceramist is done via means of two higher-order modules as in Fig. 1: (*i*) a *multiplexed-hash* component, parameterised over an arbitrary hashing operation, and (*ii*) a *blocked-state* component, parameterised over some instantiation of the AMQ interface. The multiplexed hash captures the relation between the meta-hash and the hashing operations of the basic AMQ, randomly multiplexing hashes to particular hashing operations of the sub-components. We construct a multiplexed-hash as a composition of the hashing operation *H* used by the AMQ in each of the *m* blocks, and a meta-hash function to distribute queries between the *m* blocks. The state of this structure is defined as pairing of *m* states of the hashing operation *H*, one for each of the *m* blocks of the AMQ, with the state of the meta-hash function. As such, hashing a value *v* with this operation produces a *pair* of type , where the first element is obtained by hashing *v* over the meta-hash to select a particular block, and the second element is produced by hashing *v* again over the hash operation *H* for this selected block. With this custom hashing operation, the state component of the Blocked AMQ is defined as sequence of *m* states of the AMQ, one for each block. The insertion and query operations work on the output of the multiplexed hash by using the first element to select a particular element of the sequence, and then use the second element as the value to be inserted into or queried on this selected state.

Having instantiated the data structure as described above, we proved the following abstract result about the false positive rate for blocked AMQs:

#### Theorem 10

**(Blocked AMQ False Positive Rate).** For any AMQ *A* with a false positive rate after inserting *l* elements estimated as *f*(*l*), for a multiplexed hash-function $hs$, after having inserted *l* distinct values $xs$, all unseen in $hs$, into an empty Blocked AMQ filter $bf$ composed of *m* instances of *A*, for any unseen $y \not \in xs$, the probability of a subsequent query $y \in _? bf$ for *y* returning true is given by: 

We instantiated this interface with each of the previously defined AMQ structures, obtaining the Blocked Bloom filters, Counting Blocked Bloom filters and Blocked Quotient filter along with proofs of similar properties for them, for free.

## Discussion and Related Work

*Proofs About AMQs.* While there has been a wealth of prior research into approximate membership query structures and their probabilistic bounds, the prevalence of paper-and-pencil proofs has meant that errors in analysis have gone unnoticed and propagated throughout the literature.

The most notable example is in Bloom’s original paper [6], wherein dependencies between setting bits lead to an incorrect formulation of the bound (equation (17)), which has since been repeated in several papers [9, 14, 15, 33] and even textbooks [34]. While this error was later identified by Bose *et al.* [8], their own analysis was also marred by an error in their definition of Stirling numbers of the second kind, resulting in yet another incorrect bound, corrected two years later by Christensen *et al.* [10], who avoided the error by eliding Stirling numbers altogether, and deriving the bound directly. Furthermore, despite these corrections, many subsequent papers [13, 28–30, 40, 41, 46] still use Bloom’s original incorrect bounds. For example, in Putze *et al.* [40]’s analysis of a Blocked Bloom filter, they derive an incorrect bound on the false positive rate by assuming that the false positive of the constituent Bloom filters are given by Bloom’s bound. While the Ceramist is the first development that, to the best of our knowledge, provides a mechanised proof of the probabilistic properties of Bloom filters, prior research has considered their deterministic properties. In particular, Blot *et al.* [7] provided a mechanised proof of the absence of false negatives for their implementation of a Bloom filter.

*Mechanically Verified Probabilistic Algorithms.* Past research has also focused on the verification of probabilistic algorithms, and our work builds on the results and ideas from several of these developments. The ALEA library tackles the task of proving properties of probabilistic algorithms [3], however in contrast to our deep embedding of computations, ALEA uses a shallow embedding through a Giry monad [20], representing probabilistic programs as measures over their outcomes. ALEA also axiomatises a custom type to represent reals between 0 and 1, which means they must independently prove any properties on reals they use, increasing the proof effort. The Foundational Cryptography Framework (FCF) [39] was developed for proving the security properties of cryptographic programs and provides an encoding of probabilistic algorithms. Rather than developing tooling for solving probabilistic obligations, their library proves probabilistic properties by reducing them to standard programs with known distributions. While this strategy follows the structure of cryptographic proofs, the simple tooling makes directly proving probabilistic bounds challenging. Tassarotti *et al.* ’s Polaris [47] library for reasoning about probabilistic concurrent algorithms, also uses the same reduction strategy, and thereby inherits the same issues with proving standalone bounds. Hölzl considers mechanised verification of probabilistic programs in Isabelle/HOL [27], using a similar composition of probability and computation monads to encode probabilistic programs. However, his construction defines the semantics of programs as infinite Markov chains represented as a co-inductive streams, making it unsuitable for capturing terminating programs. Our previous effort on mechanising the probabilistic properties of blockchains also considered the encoding of probabilistic computations in Coq [23]. While that work also relied on infotheo ’s probability monad, it only considered a restricted form of probabilistic properties, and did not deliver reusable tooling for the task.

*Proofs of Differential Privacy.* A popular motivation for reasoning about probabilistic computations is for the purposes of demonstrating differential privacy. Barthe *et al.* ’s CertiPriv framework [4] extends ALEA to support reasoning using a Probabilistic Relational Hoare logic, and uses this fragment to prove probabilistic non-interference arguments. More recently, Barthe *et al.* [44] have developed a mechanisation that supports a more general coupling between distributions. Given the focus on relational properties, these developments are not suited for proving explicit numerical bounds as Ceramist is.

## Conclusion

The key properties of Approximate Membership Query structures are inherently probabilistic. Formalisations of those properties are frequently stated incorrectly, due to the complexity of the underlying proofs. We have demonstrated the feasibility of conducting such proofs in a machine-assisted framework. The main ingredients of our approach are a principled decomposition of structure definitions and proof automation for manipulating probabilistic sums. Together, they enable scalable and reusable mechanised proofs about a wide range of AMQs.

## Footnotes

1 Ceramist stands for **Cer**tified **A**pproximate **M**embersh**i**p **St**ructures.

2 bf_addm is a trivial generalisation of the insertion to multiple elements.
